# The Frequency of Mediastinal Lymph Node Calcification in Sarcoidosis Patients and the Influencing Factors

**DOI:** 10.3390/medicina61010008

**Published:** 2024-12-25

**Authors:** Pelin Pınar Deniz, Pelin Duru Çetinkaya, Saida Mehdiyeva, İsmail Hanta

**Affiliations:** Department of Respiratory Disease, Cukurova University Faculty of Medicine, Yüreğir, Adana 01250, Turkey; pdeniz@cu.edu.tr (P.P.D.); smehdiyeva@cu.edu.tr (S.M.); ihanta@cu.edu.tr (İ.H.)

**Keywords:** sarcoidosis, lymph node, mediastinal nodes, hilar lymphadenopathy, calcification

## Abstract

*Background and Objectives*: This study investigates the prevalence of calcification in mediastinal lymph nodes among sarcoidosis patients and the influencing factors. Sarcoidosis is a multisystemic inflammatory disease characterized by non-caseating epithelioid granulomas. Bilateral hilar lymphadenopathy (LAP) is the most common radiographic finding, with studies showing a correlation between the frequency of lymph node calcification and disease duration, with a frequency of 3% relating to a duration of 5 years and a frequency of 20% relating to one of 10 years. *Materials and Methods*: This study involved fifty-seven patients diagnosed with sarcoidosis at our chest disease outpatient clinic from January 2020 to September 2022. We examined patient records to determine demographics, radiological findings, and respiratory function parameters. *Results*: The mean age of patients was 55.07 ± 13.53 (21–90). We identified eighty percent of patients with stage 2 sarcoidosis. Hilar lymph node calcification was observed in 13 cases (22.8%). Among the 13 cases, punctate calcification was detected in 2 (15.4%), while diffuse calcification was observed in 11 (84.6%). The mean duration of sarcoidosis diagnosis in patients was 4.1 ± 3.2 years (range: 1–14 years). Within the first 5 years after diagnosis, calcification in lymph nodes was detected in 18.6% of cases, while of patients diagnosed more than 5 years ago, 35.71% showed lymph node calcification. *Conclusions*: Our findings suggest that mediastinal lymph node calcification is more common than previously reported, and integrating clinical evaluation and patient history in cases with bilateral hilar LAP can help to avoid unnecessary invasive and costly procedures.

## 1. Introduction

Sarcoidosis is a heterogeneous disease with a variable natural course that can affect multiple organs. Sarcoidosis is a multisystemic, inflammatory disease characterized by non-caseating epithelioid granulomas of unknown etiology [1,2]. The incidence of sarcoidosis varies considerably. Annual prevalence estimates in East Asia range from 0.5 to 1.3 per 100,000 individuals, whereas in the United States and Canada, the frequency is approximately 7 to 11 per 100,000. The lungs, lymph nodes, and skin are the most commonly affected organs. Even though sarcoidosis frequently affects the lungs, detailed evaluations indicate extrathoracic manifestations upon presentation in approximately fifty percent of patients. Among the most frequently observed early extrapulmonary manifestations are skin lesions, peripheral lymph node enlargement, and ocular involvement, such as uveitis and dry eyes [3,4,5,6,7,8]. Bilateral hilar lymphadenopathy (LAP) is the most common radiographic finding [9]. Peripheral LAP, involving cervical, axillary, and inguinal nodes, is present in more than 20% of patients. The affected lymph nodes show moderate swelling and are generally painless [10]. Calcifications in lymph nodes can occur in sarcoidosis. Other granulomatous processes, such as malignancies, tuberculosis (TB), or histoplasmosis, can also cause lymph node calcifications. In sarcoidosis, calcifications may manifest as punctate, amorphous, popcorn-like, or eggshell-like [11]. Sarcoidosis, tuberculosis, and lymphomas are associated with mediastinal hilar LAP. In diagnostic positron emission tomography/computed tomography (PET/CT) scans, all of these conditions may demonstrate elevated maximum standardized uptake (SUVmax) values. Further examinations, especially invasive procedures, may present risks to the patient. This study aims to examine the characteristics of lymph node calcifications in sarcoidosis and the factors that influence their occurrence. The purpose of acquiring additional information on this topic is to prevent unnecessary and expensive inquiries. The objective of this investigation is to determine the frequency of calcifications in mediastinal lymph nodes in sarcoidosis patients, identify factors influencing this occurrence, and investigate whether there is a relationship between the duration of the disease and the presence of calcifications.

## 2. Materials and Methods

### 2.1. Study Design and Data Collection

This study was designed as retrospective, observational, and single-center research. Fifty-seven patients who were diagnosed with sarcoidosis and underwent search treatment and follow-up at our Chest Diseases outpatient clinic from January 2021 to September 2022 were included in the study.

The diagnosis of sarcoidosis was determined based on clinical presentation, the demonstration of non-caseating granulomas in one or more tissue samples, and the exclusion of other granulomatous disorders. Exclusion criteria encompassed incomplete clinical data, loss to follow-up, or a final diagnosis of other conditions such as malignancy. The study excluded patients under 18 years of age or those with known malignancies.

Patient files were investigated, and demographic data, radiological findings, respiratory function values, serum angiotensin-converting enzyme (ACE) levels, serum calcium (Ca) levels, and the duration of the disease post-diagnosis were documented. Patients were categorized into two groups according to the duration since diagnosis. Group 1 included cases taking place in the first 5 years since the diagnosis of sarcoidosis at the time of inclusion in our study. Group 2 included cases with more than 5 years since the diagnosis of sarcoidosis at the time of inclusion in our study. Both groups were compared in terms of age, gender, characteristics of lymphadenopathies, blood Ca, ACE levels, and respiratory function parameters.

Ethical approval was provided for the investigation. All processes were conducted in accordance with the principles stated in the Declaration of Helsinki. The study protocol was approved by the Ethical Committee of Cukurova University School of Medicine and the study was conducted in accordance with the approved guidelines (142/39-8 March 2024).

### 2.2. Statistical Method

The SPSS 20 software program was used for data analysis. The Kolmogorov–Smirnov test was employed as the normality test. Parametric tests were preferred when the assumption of normal distribution was met, while non-parametric tests were used when it was not. For the analysis, the independent T-test was applied for normally distributed data between two independent groups, and the Pearson chi-square test was used for the analysis of nominal data. A *p* value of less than 0.05 was considered statistically significant.

## 3. Results

**Figure 1:**
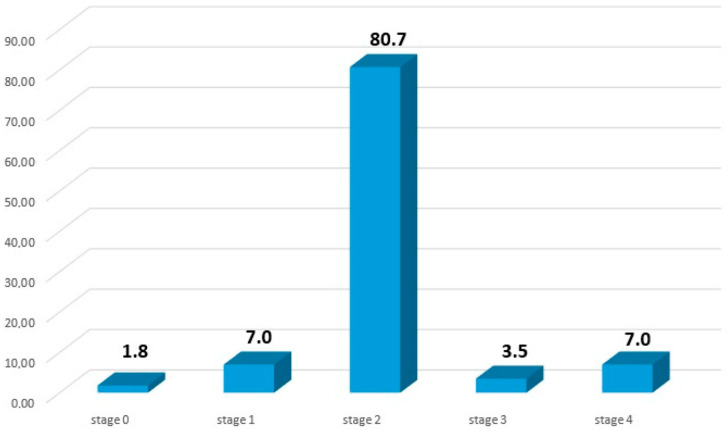
Distribution percentages of sarcoidosis cases according to disease stages.

The mean age of 57 cases is 55.07 ± 13.53 years (range: 21–90). Among the cases, 17 are male (29.8%) and 40 are female (70.2%). A smoking history was determined in 17.5% of the cases. A history of tuberculosis was observed in 7% of the cases. Comorbidities were present in 53.6% of the cases, with diabetes mellitus being the most prevalent. The cases exhibited various degrees of organ involvement, including hilar LAP (93%), lung involvement (84.2%), extrathoracic lymph node involvement (45.6%), liver involvement (33.3%), spleen involvement (12.3%), eye involvement (10.5%), skin involvement (8.8%), bone involvement (3.5%), lacrimal gland involvement (1.8%), and muscle involvement (1.8%). Among the cases, 80.7% were in stage 2 of sarcoidosis, and the distribution of cases according to stages is shown in Figure 1.

The mean values, standard deviations, and minimum–maximum ranges of serum ACE, blood Ca, urinary Ca, and respiratory function parameters for the cases are presented in Table 1. Hilar lymph node calcification was observed in 13 cases (22.8%). Among the 13 cases, punctate calcification was detected in 2 (15.4%), while diffuse calcification was observed in 11 (84.6%). The mean duration for the diagnosis of sarcoidosis in patients was 4.1 ± 3.2 years (range: 1–14 years). Within the first 5 years after diagnosis, calcification in lymph nodes was detected in 18.6% of cases, while in those diagnosed more than 5 years ago, 35.71% showed lymph node calcification.

Group 1 comprised 31 women and 12 men, while Group 2 included 9 women and 5 men. The mean age was determined to be 54.79 years in Group 1 and 55.92 years in Group 2. No statistically significant differences were observed between the groups regarding age, serum Ca, serum ACE, urinary Ca, or pulmonary function parameters (Table 2).

The ages, genders, serum Ca levels, serum ACE levels, urinary Ca levels, and respiratory function parameters of patients were examined with and without lymph node calcification. In the cohort with lymph node calcification, the serum Ca level was determined to be 9.86 ± 1.05 mg/dL, even though it was 9.91 ± 0.78 mg/dL in the group without calcification. No statistically significant differences were detected between lymph node calcification and age, gender, serum Ca, serum ACE, urinary Ca, respiratory function parameters, or disease stage.

## 4. Discussion

Nearly 150 years after its initial description, sarcoidosis still lacks standard methods for diagnosis, treatment, and monitoring [12]. In the context of sarcoidosis with mediastinal LAP, it is essential to rule out cases that do not require treatment for TB and lymphoma. In our study, calcification was detected in lymph nodes in 22.8% of cases. Within the first 5 years after diagnosis, calcification was detected in lymph nodes in 18.6% of cases, while in those diagnosed more than 5 years ago lymph node calcification was observed in 35.71% of cases. It was determined that the probability of calcification increased as the duration of sarcoidosis disease prolonged. Mediastinal lymph nodes are not only involved in localized inflammatory diseases or primary lymphatic tumors but also in primary tumors originating from the thorax or distant organs. Consequently, a wide range of diseases is responsible for the development of mediastinal LAP. The most common diseases include lymphoma, metastatic carcinoma, sarcoidosis, and TB. It is essential to distinguish benign lymph nodes from malignant ones. Unfortunately, PET-CT, typically utilized to differentiate between benign and malignant diseases, is ineffective in distinguishing benign conditions. Kumar et al. reported an SUVmax value of 11.8 in cases of TB and one of 10.9 in sarcoidosis cases.

The mean SUVmax values were 5.3 for TB and 4.6 for sarcoidosis, with no statistically significant difference detected between the two groups [13]. Additionally, elevated SUVmax values have been documented in sarcoidosis and tuberculosis, similar to malignant conditions. Teirstein et al. reported an SUVmax value of 15.8 for sarcoidosis [14]. The elevated SUV max values identified using PET-CT require clinicians to perform additional investigations through invasive procedures for diagnosis, such as endobronchial ultrasound, endoscopic ultrasound-guided fine-needle aspiration, and mediastinoscopy. In our country, sarcoidosis is the most commonly observed among interstitial lung diseases, with an estimated annual incidence of 4 per 100,000 people, consistent with studies conducted in Europe [15,16]. Additionally, the incidence of TB in our country has been reported to be 14.1 per 100,000 [17]. Therefore, we meet difficulties in the differential diagnosis of benign mediastinal lymph node disorders in our pulmonology outpatient clinics. In both sarcoidosis and tuberculosis cases, PET-CT may demonstrate SUV uptake, and lymph node calcification can be observed in both groups. Lymph node calcification is observed in sarcoidosis patients; however, there is unfortunately insufficient literature regarding the rates of calcification and the factors influencing it. Calcified hilar lymph nodes may occur in approximately 5% of sarcoidosis cases and serve as a sign of longstanding and chronic sarcoidosis [18]. In our study, mediastinal lymph node calcification was detected in 22.8% of cases, which is a higher rate than reported in the literature. While the presence of necrotic and heterogeneous-appearing LAPs increases the likelihood of malignancy, the presence of calcification raises the possibility of a benign condition. Necrosis in the center of the lymph node and ring-like staining are almost diagnostic for tuberculous lymphadenitis. In lymphoma, nodal calcification is not expected prior to treatment; however, in rare cases, particularly with untreated nodes in non-Hodgkin’s lymphoma, calcification may be observed in about 8% of cases. In sarcoidosis, the radiologically typical features of lymph nodes are as follows: frequent calcification, well-defined nodules, hilar and mediastinal involvement of equal weight (lambda sign), and bilateral and symmetrical hilar involvement. Gawne-Cain et al. examined 77 patients with lymph node calcification and noted that bilateral hilar calcification is more common in sarcoidosis. They reported that focal calcification is frequently observed in sarcoidosis, while diffuse calcification is typically seen in tuberculosis [19]. In our study focusing on sarcoidosis patients, focal calcification was observed in 15.4% of patients with calcification, while diffuse calcification was noted in 84.6%. The literature has reported calcifications resembling eggshell calcifications. In the study by Israel et al., it was reported that the frequency of lymph node calcification in sarcoidosis was directly proportional to the duration of the disease; calcification develops in 3% of patients within 5 years and in 20% within 10 years. This study indicated that in 111 sarcoidosis patients followed for 10 years or longer, calcification developed in more than 20% of mediastinal lymph nodes [20]. In our study, the average duration from the diagnosis of sarcoidosis to the detection of lymph node calcification was found to be 4.1 ± 3.2 years. Among the cases, calcification was observed in 18.6% within the first 5 years following diagnosis, while calcification was detected in 35.71% of cases diagnosed more than 5 years ago. Consistent with the literature, it appears that the calcification of mediastinal lymph nodes may become more prevalent as the duration of the disease increases. Although it is known that calcification may occur in lymph nodes in sarcoidosis cases, the reasons for calcification and the factors influencing it remain unclear. In Scadding’s article, which proposed hypotheses regarding hypercalcemia in sarcoidosis, no relationship was found between hypercalcemia and the development of calcification [21]. The literature does not contain any studies that investigate the factors that induce calcification in lymph nodes in sarcoidosis or the factors that influence them. We analyzed gender, age, disease stage, serum calcium, serum ACE, urinary calcium levels, and respiratory function parameters to determine the factors influencing calcification in sarcoidosis among groups with and without mediastinal lymph node calcification; however, no statistically significant correlation was identified between the groups.

Our study has some limitations. Although stage 1 sarcoidosis is common, the higher prevalence of stage 2 sarcoidosis in our study can be attributed to our status as a tertiary care hospital and the inclusion of patients undergoing treatment and follow-up. Consequently, our calcification rate may also appear elevated. Another limitation of our study is that the distribution of sarcoidosis patient groups was not equal because the patients diagnosed and followed up in our hospital were selected within a specific time period.

## 5. Conclusions

Our study has shown that mediastinal lymph node calcification is observed more frequently than is reported in the literature. By supporting cases with bilateral hilar LAP and the calcification of mediastinal lymph nodes through clinical evaluation and the assessment patient history, we can protect patients from unnecessary invasive and costly diagnostic procedures.

Unfortunately, for patients with mediastinal LAP detected radiologically, a PET-CT is often requested, and since SUV uptake can also be high in sarcoidosis, invasive procedures are performed. Both PET-CT and the invasive procedures not only increase costs but can also pose risks to the patient. In our study, due to the high rates of calcification in sarcoidosis, we believe that before performing a PET-CT on every patient with mediastinal LAP, a clinical and radiological evaluation should be conducted, and if there are no risk factors for malignancy, follow-up protocols may be applied. This article can serve as a guide for clinicians in this context.

## Figures and Tables

**Table 1:** The mean values and standard deviations of the respiratory function parameters, serum ACE, calcium, and urinary calcium values for the cases.

Laboratory Values	Mean ± Standard Deviation (Min-Max)
ACE (microgram/L)	65.98 ± 109.38 (9–335)
Urine calcium (mg/dL)	12.61 ± 7.6 (2.30–28.65)
Calcium (mg/dL)	9.90 ± 0.83 (8.74–12.58)
Respiratory function parameters	
FCV(%)	101.38 ± 15.92(63–137)
FEV1(%)	94.61 ± 16.57 (60–132)
FEV1/FVC	77.89 ± 6.07 (61.76–89)
DLCO	71.32 ± (22–118)

Mean (M), standard deviation (SD), minimum (Min), and maximum (Max) values of laboratory and respiratory function parameters in cases. FCV: forced vital capacity; FEV1: forced expiratory volume in 1 s; DLCO: diffusing capacity of the lung for carbon monoxide; ACE: angiotensin-converting enzyme.

**Table 2:** The mean values and standard deviations of the respiratory function parameters, serum ACE, calcium, and urinary calcium values for Group 1 and Group 2.

	Grup 1 (*n* = 43)	Grup 2 (*n* = 14)
Male (*n* = 17) Female (*n* = 40)	12 31	5 9
Age (Mean ± SD)	54.79 ± 14.22	55.92 ± 11.57
DLCO (Mean ± SD)	69.30 ± 25.45	77.12 ± 23.58
FEV1 (Mean ± SD)	94.73 ± 17.66	94.25 ± 13.66
FVC (Mean ± SD)	101.83 ± 17.15	100 ± 12.16
FEV1/FVC (Mean ± SD)	77.89 ± 6.59	77.89 ± 4.42
Calcium (mg/dL) (Mean ± SD)	9.78 ± 0.71	10.31 ± 1.08
Urine calcium (mg/dL) (Mean ± SD)	12.60 ± 9.25	12.63 ± 1.83
ACE (microgram/L) (Mean ± SD)	65.98 ± 109.38	66.88 ± 100.8

Mean (M), standard deviation (SD), minimum (Min), and maximum (Max) values of laboratory and respiratory function parameters in cases. FCV: forced vital capacity; FEV1: forced expiratory volume in 1 s; DLCO: diffusing capacity of the lung for carbon monoxide; ACE: angiotensin-converting enzyme.

## Data Availability

The datasets used and analyzed during the current study are available from the corresponding author on reasonable request.
